# Enhanced antibacterial and anticancer activities of plant extract mediated green synthesized zinc oxide-silver nanoparticles

**DOI:** 10.3389/fmicb.2023.1194292

**Published:** 2023-07-24

**Authors:** Siti Nur Amalina Mohamad Sukri, Kamyar Shameli, Sin-Yeang Teow, Jactty Chew, Li-Ting Ooi, Michiele Lee-Kiun Soon, Nur Afini Ismail, Hassan Moeini

**Affiliations:** ^1^Malaysia-Japan International Institute of Technology, Universiti Teknologi Malaysia, Kuala Lumpur, Malaysia; ^2^School of Medicine, Institute of Virology, Technical University of Munich, Munich, Germany; ^3^Department of Biology, College of Science, Mathematics and Technology, Wenzhou-Kean University, Wenzhou, Zhejiang, China; ^4^Department of Biological Sciences, School of Medical and Life Sciences, Sunway University, Bandar Sunway, Selangor, Malaysia; ^5^School of Health Sciences, International Medical University, Kuala Lumpur, Malaysia; ^6^Department of Medical Sciences, School of Medical and Life Sciences, Sunway University, Bandar Sunway, Selangor, Malaysia

**Keywords:** zinc oxide, silver, nanoparticles, green synthesis, *Punica granatum*, antibacterial, anticancer

## Abstract

This study presents a green synthesis approach for the fabrication of zinc oxide-silver nanoparticles (ZnO-Ag-NPs) using *Punica granatum* fruit peels extract as a natural reducing and stabilizing agent. This eco-friendly method offers a sustainable alternative to conventional methods that often employ toxic or hazardous chemicals. Antibacterial and anti-cancer activities of the green synthesized nanoparticles were then assessed *in vitro*. X-ray diffraction confirmed the production of ZnO-Ag-NPs with increasing crystallinity in higher pH values. The ZnO-Ag-NPs were found to be agglomerated with spherical Ag-NPs. Fourier Transform Infrared (FTIR) spectra revealed a broad band in ZnO-Ag-NPs ranging from 400^−1^ to 530 cm^−1^ with reduced intensity as compared to ZnO-NPs, indicating the formation of Ag-NPs on the surface of ZnO-NPs. The synthesized ZnO-Ag-NPs exhibited potent antibacterial activity against a broad spectrum of bacterial strains, particularly Gram-positive bacteria, with superior inhibition activity compared to ZnO-NPs. Moreover, ZnO-Ag-NPs showed a dose-dependent anti-proliferative effect on colorectal-, lung-, and cervical cancer cells. ZnO-Ag-NPs showed significantly greater efficacy in inhibiting cancer cell growth at a lower concentration of 31.25 μg/mL, compared to ZnO-NPs which required over 500 μg/mL, possibly due to the presence of silver nanoparticles (Ag-NPs). The results obtained from this study demonstrate the potential of green synthesis approaches in the fabrication of therapeutic nanomaterials for cancer treatment, as well as other biomedical applications.

## 1. Introduction

For several decades now, nanotechnology has emerged as a promising technology in various fields such as biomedical sciences, energy production, nano-electronics and consumer products (Rajendran and Mani, 2020). Nanoparticle-based therapy has been suggested as an alternative option to resolve some of world’s pressing problems such as antimicrobial resistance (AMR) and cancer. AMR occurs when a microorganism develops natural resistance to an antimicrobial drug that was originally effective for the treatment of infections caused by it (Mariappan et al., 2021). These drug-resistant microbial strains or ‘superbugs’, are extremely difficult to treat with limited existing antimicrobial agents. Without proper actions to combat this issue, it is estimated that AMR will cost up to 10 million lives by the year of 2050 with more that 4 million deaths in Asia continent alone (Shankar, 2016). Therefore, in an effort to address this extremely concerning matter, World Health Organization (WHO) has urged researchers all around the world for the research and development of novel antimicrobial agents, in particular antibacterial drugs (Sánchez-López et al., 2020).

Another major disease and leading cause of deaths that has long been burdening the human population is cancer. Cancer is a prominent illness triggered by genetic mutations in cellular DNA, damaging the mechanism regulating cell death and cell division, leading to uncontrolled proliferation of cells in human body (Doughty et al., 2019). Conventional cancer treatments such as surgery, radiotherapy and chemotherapy are effective, however, they come with severe side effects that reduce patients’ quality of life. In addition, patient can also develop resistance to various chemotherapeutic drugs, a phenomenon known as multidrug resistance (MDR) causing significant obstacles in treating cancer (Abdalla et al., 2018). Innovation of new modern treatment by using nanotechnology can help improve the shortcomings of current cancer therapies.

Amongst different types of nanomaterials, metallic nanoparticles (NPs) are well known to offer special qualities such as enhanced properties due to high surface-to-volume ratio, increased mobility because of their small size and bioavailability as they can be used for specific drug targeting (Salem, 2023; Shehabeldine et al., 2023). There is a wide range of metallic NPs such as titanium dioxide (TiO_2_), iron oxide (Fe_3_O_4_), zinc oxide (ZnO), copper (Cu), gold (Au), silver (Ag), platinum (Pt), nickel (Ni) and many more (Yang et al., 2008; Ahmed and Ikram, 2016; Sukri et al., 2019; Iqbal et al., 2020; Izadiyan et al., 2020; Vu et al., 2020; Ismail et al., 2021). One of the most extensively studied metal oxide NPs is ZnO due to its many advantages (Mohamed et al., 2021; Abdelaziz et al., 2022; Abdelghany et al., 2023). ZnO is a renowned inorganic material that has been widely used in everyday applications. Compared to other metal oxides, ZnO is comparably inexpensive and deemed non-toxic since the Food and Drug Administration (FDA) has listed it as a generally recognized as safe (GRAS) material (Espitia et al., 2012). Based on previous studies, ZnO in nanoscale perform considerably well in various biomedical applications and drug delivery systems due to its biocompatibility nature (Sadhasivam et al., 2021). Despite promising reports of ZnO-NPs efficiency in nanomedicine, there are still intrinsic limitations such as UV-dependent optical absorption, instability in biological fluids and unpredictable cytotoxic effects that need to be improved (Carofiglio et al., 2020). To overcome these limitations and to further boost its biological activities, in the present study, we coupled ZnO-NPs with Ag-NPs. Ag-NPs have been shown to have superior properties in biomedical studies especially antimicrobial applications (Faried et al., 2016; Wan-Mat-Khalir et al., 2020a,b). Ag-NPs can inhibit the growth of a wide range of Gram negative and Gram positive bacteria (Abinaya et al., 2016; Nguyen et al., 2019). They have also reported to have cytotoxic activities against different types of cancer cells (Shankar, 2016; Ismail et al., 2019).

This past decade, the biosynthesis of NPs using green synthesis methods has emerged as a promising alternative to conventional chemical and physical methods. These green synthesis methods have garnered attention as they are more ecofriendly, sustainable and cost-effective. Previous publications have also reported that biosynthesized NPs exhibited high antioxidant as well as antibacterial efficacy (Alyamani et al., 2021). The present research aimed to investigate the synthesis of ZnO-Ag-NPs using *Punica granatum* fruit (*P. granatum* F.) peels extract as a reducing and capping agent in the fabrication process. *P. granatum* F. peels extract contains a substantial amount of beneficial phytochemical compounds that play a key role during the synthesis process of the NPs. This study evaluated the physicochemical properties of the NPs, particularly focusing on the effects of varying pH conditions on the ZnO-Ag-NPs synthesis. Furthermore, the antibacterial and cytotoxic effects of the NPs were assessed against different bacterial strains and cancer cells, providing valuable insights whether incorporation of Ag with ZnO-NPs enhances their effectiveness.

## 2. Materials and methods

### 2.1. Chemicals, bacterial strains and cell lines

All metal salt precursors used for biosynthesis of ZnO-NPs and ZnO-Ag NPs were of analytical grade. Zinc nitrate hexahydrate (Zn(NO_3_)_2_.6H_2_O, 98%), silver nitrate (AgNO_3_, 98%) and ammonia solution (NH_4_OH, 30%) were purchased from R&M Chemicals, United Kingdom. For antibacterial experiment, three Gram-positive (*Staphylococcus aureus* ATCC 25923, methicillin-resistant *Staphylococcus aureus* clinical isolate and *Bacillus subtilis* ATCC 6633) as well as three Gram-negative strains (*Escherichia coli* ATCC 11775, rifampicin-resistant *Escherichia coli* K1 clinical isolate and *Salmonella enterica* ATCC 14028) were tested. Human colorectal carcinoma cell (HCT116: ATCC CCL-247), lung cancer cells (A549) and immortalized cervical cancer cells (Hela) were used for *in vitro* cytotoxicity assay. The cell lines were maintained in Dulbecco’s Modified Eagle’s medium (DMEM) supplemented with 10% fetal bovine serum (FBS) (Gibco) and 100 U/mL penicillin/streptomycin (Gibco).

### 2.2. Fruit peels extract preparation

Fresh fruit peels of *P. granatum* were collected from the “Green Farm” in Fars province, Neyriz, Iran. Plant extract was prepared as previously described (Sukri et al., 2019). Briefly, fresh *P. granatum* F. peels were dried ground into fine powder and then were subjected to aqueous extraction with distilled water at 60°C for 60 min. The crude extract was clarified by filtration followed by centrifugation at 4000 rpm for 10 min, and kept at 4°C for further experiments.

### 2.3. Synthesis of ZnO-NPs and ZnO-Ag-NPs

ZnO-NPs were fabricated using the same sol–gel and combustion procedure as previously described (Sukri et al., 2019). Concisely, zinc nitrate hexahydrate and the fruit peels extract were stirred at 90°C until a gel-like product was formed. After annealing process at 600°C for 60 min, ZnO-NPs, namely Z-6, were produced, in the form of a fine white colored powder. The NPs were then used for the biosynthesis of ZnO-Ag-NPs by precipitation method. For this aim, *P. granatum* F. peels extract and 10 mL, AgNO_3_ (0.1 M) were added to 0.1 g of ZnO-NPs followed by continuously stirring in a beaker. Ammonium hydroxide (1 M) solution was added drop by drop into the beaker to reach to the desired pH level (pH 4, 7, and 9). After 60 min’ incubation at 55°C in a water bath shaker, the solutions were centrifuged at 10,000 rpm for 10 min. The precipitates were then washed three times with water and ethanol. Finally, the samples were dried at 60°C in an oven. The resulting samples are named as ZA-4, ZA-7, and ZA-9 to represent ZnO-Ag-NPs synthesized at pH 4, 7, and 9.

### 2.4. Characterization methods and instrumentation

The crystalline phase of all samples was investigated using X-ray diffraction (X’Pert, Philips, Netherlands) using Cu Kα radiation at the angle range of 2θ (10–90°). Ultraviolet–visible (UV 1800, SHIMADZU, Japan) spectroscopy (UV-vis) was utilized in the range of 300 to 700 nm to observe the absorption peaks of each biosynthesized sample. Fourier Transform Infrared Spectroscopy analysis (FTIR Model 1650, PerkinElmer, USA) was carried out in the range of 400–4000 cm^−1^ wavenumber to detect functional groups on the NPs. Potassium bromide (KBr) method was adopted to analyze ZnO and ZnO-Ag-NPs, while attenuated total reflection (ATR) method was used for *P. granatum* F. peels extract. The surface morphology and electron diffraction pattern of ZnO and ZnO-Ag-NPs were investigated using Transmission Electron Microscopy (TEM) with Energy Dispersive X-Ray Spectroscopy (EDS, JEM-2100F, Jeol Ltd., Japan).

### 2.5. Antibacterial studies

Broth micro-dilution method was used to determine the minimum inhibitory concentration (MIC) of the NPs toward bacterial strains using the Clinical and Laboratory Standards Institute (CLSI) protocols as previously described (Ismail et al., 2019; Sukri et al., 2019). To this end, single colonies of fresh bacterial cultures on Mueller Hinton agar (MHA) plates were inoculated into sterile Mueller Hinton broth (MHB) for overnight culture (12–18 h) at 37°C prior to the experiments. Bacterial concentration was then standardized to an optical density (OD) of 600 nm (approximately 10^8^ CFU/mL) with MHB. Two-fold serial dilutions of NPs were prepared in 96-well plates to give the final test concentrations of 0, 7.8, 15.6, 31.3, 62.5, 125, 250, and 500 μg/mL per well. Ten μl of bacterial suspension equivalent to 10^6^ CFU/mL of exponentially growing bacterial cells were added to the wells. After 18 h incubation at 37°C, OD 600_nm_ was determined using microplate reader (Tecan), and MIC_50_ was then measured using an online calculator.^1^ Three independent experiments were performed and the data are expressed as the mean ± standard deviation for all triplicates within an individual experiment.

### 2.6. Cytotoxicity assay

Cell Titer-Glo 2.0 Cell Viability Assay (#G9241, Promega) was used to determine the cytotoxic effect of NPs on HCT116 colorectal, A549 lung cancer cells and Hela cells according to the manufacturer’s instruction (Yusefi et al., 2021). Briefly, 5 × 10^3^ cells were seeded in 96-well plate (100 μL/well) and incubated overnight at 37°C in a 5% CO_2_, 95% humidified incubator. The next day, two-fold serially diluted samples at the concentrations of 0–500 μg/mL (100 μL/well) were added into the cells. After 72 h’ incubation at 37°C in a 5% CO_2_ incubator, 20 μL of the MTS reagent was added into the wells and incubated for an additional 3 h at 37°C in a 5% CO_2_ incubator. The optical density (OD) was then measured at 490 nm using a multimode microplate reader (Tecan). The dose–response graph was plotted by calculating the percent cell viability using an equation below:


$$Percentcellviability\;\left(\%\right)=\frac{mean\;OD\;ofsamplewell}{mean\;OD\;ofcontrolwell}\;X\;100\;\left(1\right)$$

Inhibitory concentration causing 50% growth inhibition (IC_50_) was determined using an online calculator. Three independent experiments were performed and the data are expressed as the mean ± standard deviation for all triplicates within individual experiments.

### 2.7. Statistical analysis

The cytotoxicity and antibacterial data was analyzed by *t*-test; statistical significances were set at *p* < 0.05, the results were expressed as means ± standard deviation and the graphs were drawn by GraphPad Prism 9.5.0. The histogram of particle size distribution, which was sized according to the TEM images and by the ImageJ-Fiji software, was drawn by SPSS 16.0 software.

## 3. Results and discussion

Previously, *P. granatum* F. peels extract has been reported as a successful reducing and stabilizing agent in the production of individual ZnO-NPs and Ag-NPs (Joshi et al., 2018; Sukri et al., 2019). A plethora amount of phenolic compounds is mainly concentrated in the peels portion of *P. granatum* F. making it a highly valuable resource in this research. Based on an extensive study by Singh et al. (2018) three major phenolic compounds were found in *P. granatum* F. peels extract; phenolic acids, flavonoids and tannins. Examples of phenolic acids include chlorogenic, caffeic, syringic, sinapic, p-coumaric, vanillic, ellagic, gallic acid and cinnamic acid (Sarker and Oba, 2020). Under flavonoids, phytochemicals such as catechin, epicatechin, quercetin, anthocyanins and procyanidins were found in the peels extract. Meanwhile, major tannins compound extracted from *P. granatum* F. peels are ellagitannins with punicalagin and punicalagin derivatives (Abid et al., 2017; Singh et al., 2018).

Bioactive compounds in the extract, specifically polyphenols including tannins, ellagic and gallic acid have been suggested to play an important role in the conversion of silver ions (Ag^+^) to silver atoms (Ag^0^) (Ismail et al., 2019; Magangana et al., 2020). Polyphenols comprise of two or more hydroxyl groups located in *ortho*- or *para*- position of their phenyl rings which can be easily oxidized to quinone (Spiegel et al., 2020). It is assumed that interaction between Ag^+^ and quinone groups in the peels extract can stabilize the particles and affect the size of the nanoparticles (Yıldırım et al., 2013).

**Figure 1:**
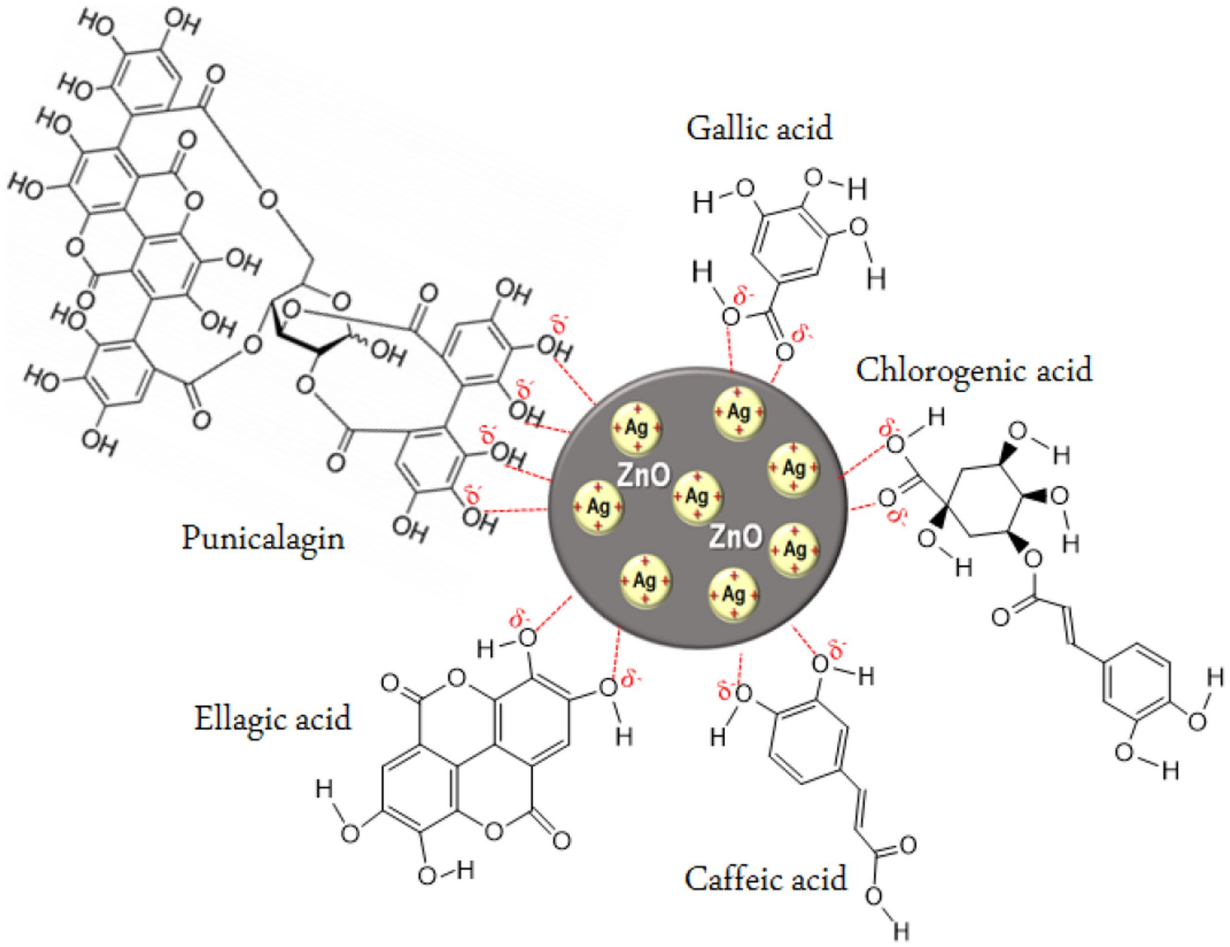
Proposed illustration for the interaction of silver ions with phytochemical compounds in *P. granatum* F. peels extract.

In the present study, we synthesized ZnO-Ag-NPs through precipitation method in the presence of *P. granatum* F. peels extract. Figure 1, shows the schematic illustration for possible interactions between Ag^+^, in the silver precursor, and bioactive compounds in the *P. granatum* F. peels extract in the process of hybrid ZnO-Ag-NPs production. It seems that negatively charged atoms in the major compounds of the peel extract donate their electrons to assist in the reduction process of positively charged Ag^+^ on the surface of ZnO-NPs. In terms of ZnO-Ag-NPs stability, previous publications have reported negative zeta potential values between −13 and −27 mV with high stability for over 3 months, owing to the electrostatic repulsion forces of the negatively charged particles (Das et al., 2016; Rajendran and Mani, 2020; Mirzaee-Rad et al., 2023).

Biosynthesis of ZnO-Ag-NPs, namely as ZA-4, ZA-7 and ZA-9, was carried out at acidic (pH 4), neutral (pH 7) and alkaline (pH 9) conditions, respectively, and their physical and chemical properties were investigated and compared with the pristine ZnO-NPs, named as Z-6.

As a result, if 0.1 g of ZnO-NPs is mixed with 10 mL, AgNO_3_ (0.1 M), the theoretical yield of ZnO-Ag-NPs will be 0.269 g. It is important to note that this is a theoretical yield, and the actual yield may be different due to various factors such as incomplete reaction, loss during isolation, or impurities. So, since the experimental weight of the ZnO-Ag-NPs for ZA-4, ZA-7 and ZA-9 were 0.198 g, 0.234 g and 0.253 g respectively, the yield of reaction was 73.61, 86.98, and 94.05%. As a result, by increasing the pH of the solutions, the reaction efficiency of the preparation of Ag-NPs loaded on ZnO-NPs increased.

### 3.1. X-ray diffraction analysis (XRD)

**Figure 2:**
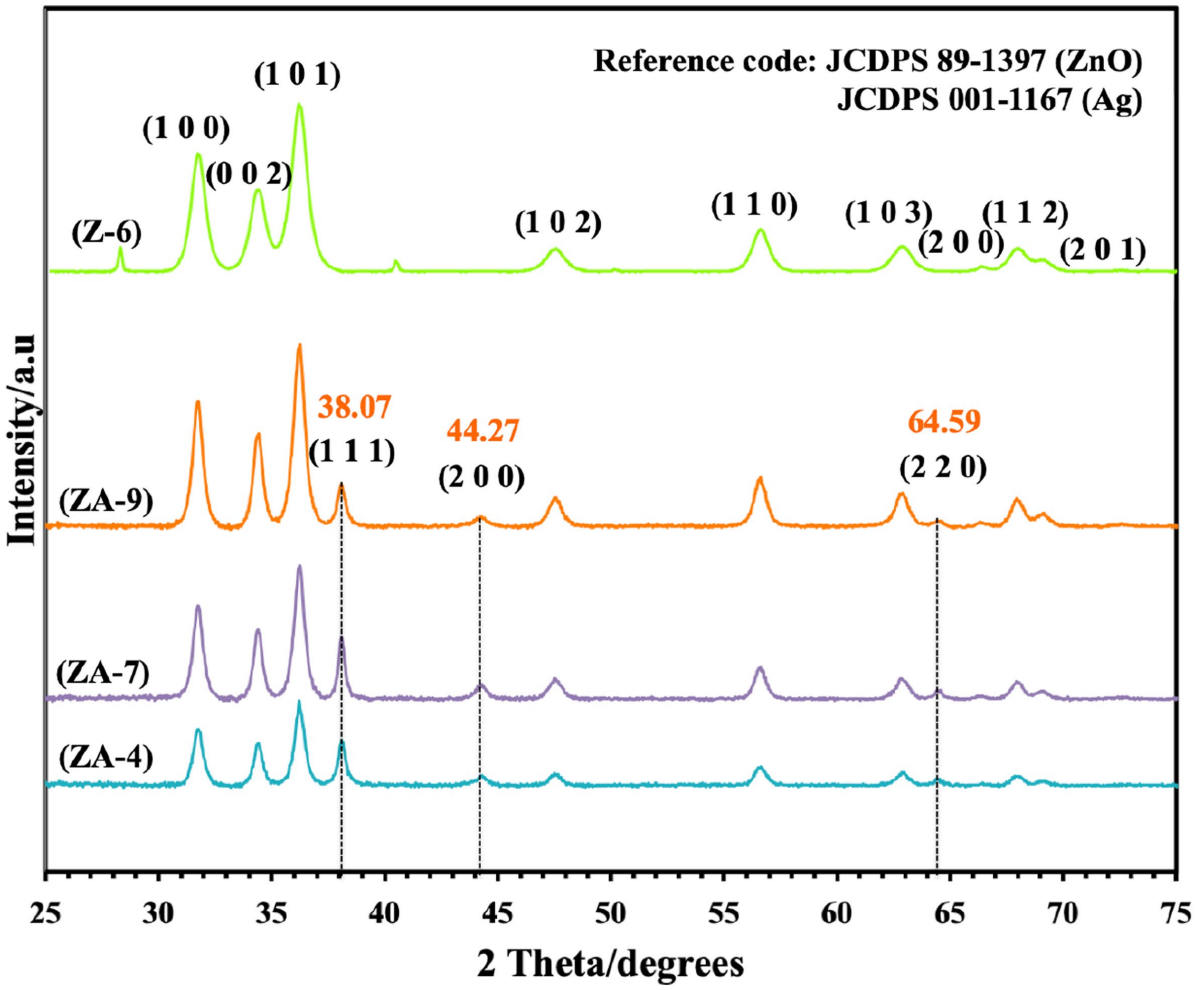
X-ray diffraction graph of ZnO (Z-6) and ZnO-Ag-conjugated (ZA-4, ZA-7, and ZA-9) nanoparticles.

Successful synthesis of ZnO-NPs and ZnO-Ag-NPs was confirmed by XRD. As shown in Figure 2, the pristine ZnO-NPs (Z-6) revealed narrow and intense peaks, indicating high crystallinity of the nanoparticle; the diffraction peaks showed 2θ values of 32.00°, 34.66°, 36.48°, 47.77°, 56.76°, 63.06°, 66.53°, 68.13°, and 69.27° corresponding to the crystal planes of (100), (002), (101), (102), (110), (103), (200), (112), and (201), respectively. These peaks are indexed to the ZnO hexagonal wurtzite phase structure supported by JCDPS Cardno. 89-1397 data (Karaköse et al., 2017). No other peaks related to any foreign compounds were detected in the Z-6 samples.

Similar ZnO peaks were detected in the ZnO-Ag-NPs (ZA-4, ZA-7 and ZA-9), confirming the successful formation of the crystalline structures. Ag diffraction peaks were represented with 2θ values of 38.07°, 44.42°, and 64.59 corresponding to the crystal planes of (111), (200), and (220), respectively. These peaks can be indexed to face centered cubic phase metallic silver supported by JCDPS Cardno. 001-1167 (Basavalingiah et al., 2019). The absence of other diffraction peaks verifies the successful fabrication of ZnO-Ag-NPs with *P. granatum* F. peels extract as a mediating solvent. The diffraction peaks were found to be narrower and more intense for ZA-9 as compared to ZA-4 and ZA-7, which might refer to the increased crystallinity of ZA-9 in basic environment.

### 3.2. UV-visible spectroscopy analysis

**Figure 3:**
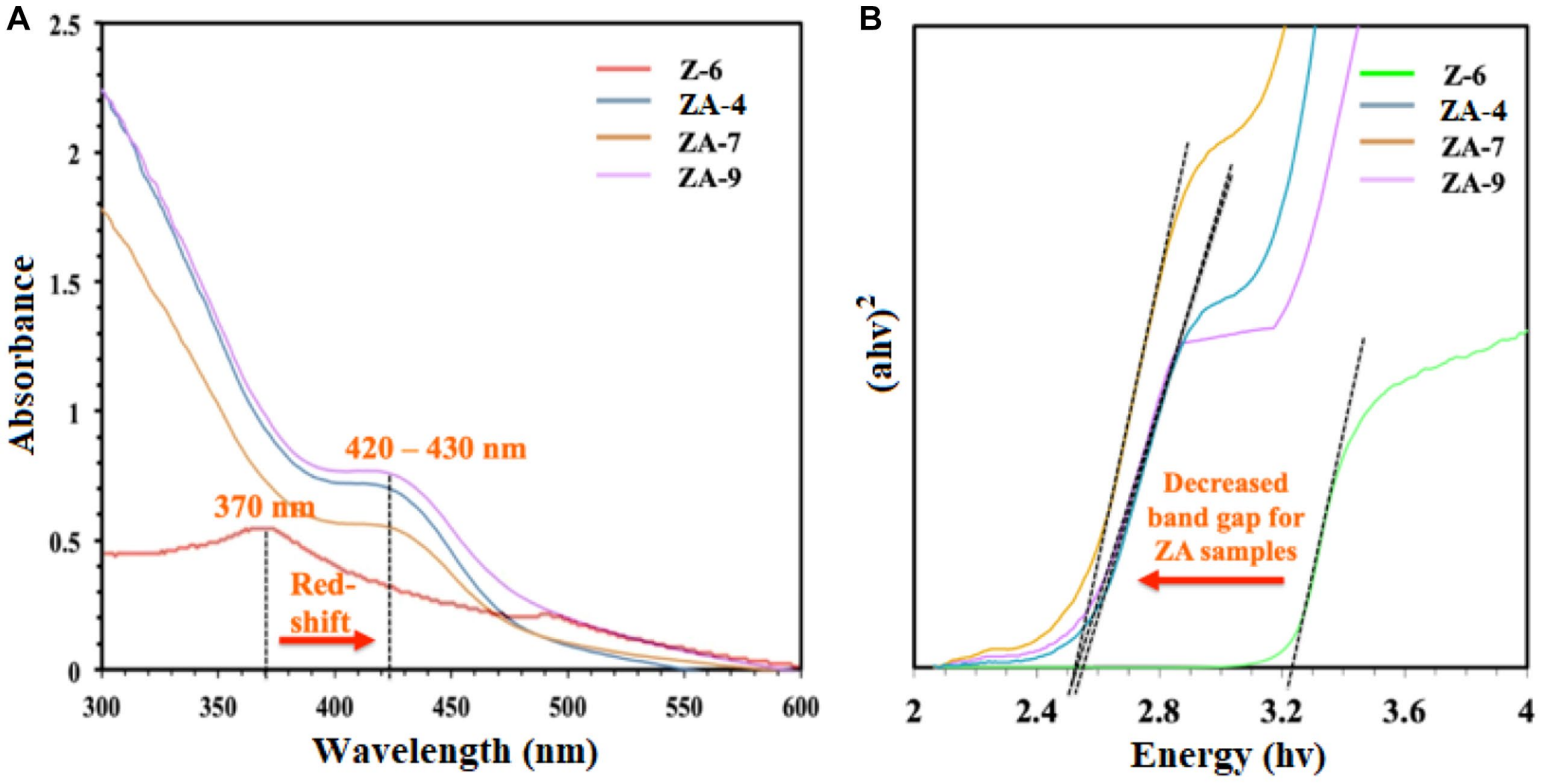
**(A)** UV-vis spectra and **(B)** Tauc’s plot for the ZnO- (Z-6) and ZnO-Ag-NPs (ZA-4, ZA-7, and ZA-9).

UV-vis spectroscopy was also used to confirm the biosynthesis of the NPs. As shown in Figure 3A, compared to ZnO-NPs (Z-6), the ZnO-Ag-NPs revealed a shift in the excitonic absorption peaks from 370 nm toward longer wavelength at 420–430 nm (red-shift) due to localized surface plasmon resonance (LSPR). This shift indicates successful Ag modification through substitution of Ag^+^ ions into the Zn^2+^ sites on the ZnO lattice, resulting in band-gap energy changes (Zare et al., 2019). To calculate the optical band-gap value of all ZnO-Ag-NPs samples, Tauc’s plot was plotted, where a significant decrease in band-gap value was observed in ZnO-Ag-NPs samples when compared to the pristine ZnO-NPs, as presented in Figure 3B. The band-gap value was projected to be 2.541, 2.485, and 2.515 eV for the ZA-4, ZA-7, and ZA-9, respectively. Higher band-gap value (3.283 eV) was detected for the Z-6.

### 3.3. Fourier transform infrared spectroscopy analysis (FTIR)

**Figure 4:**
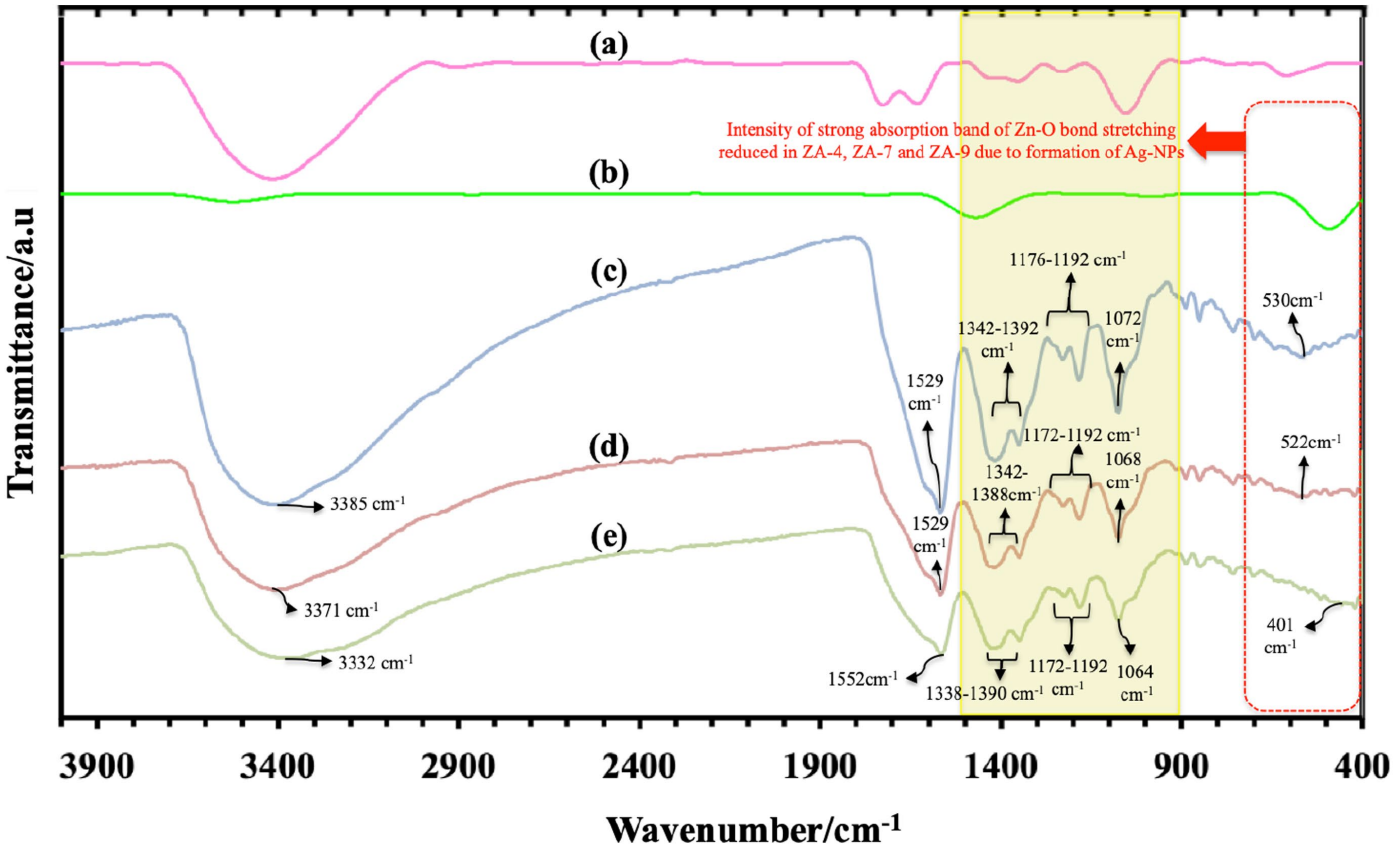
Fourier transform infrared spectroscopy spectra of *P. granatum* peel extract **(A)**, and the synthesized nanoparticles: Z-6 **(B)**, ZA-4 **(C)**, ZA-7 **(D)**, and ZA-9 **(E)**.

FTIR spectroscopy data analysis, as shown in Figure 4, determined functional groups in ZnO-NPs and all ZnO-Ag-NPs. Characteristic peaks of hydroxyl groups, due to atmospheric moisture absorption, were detected between 3332 and 3385 cm^−1^. This stretching mode of O-H groups can be due to the formation of inter- and intra-molecular hydrogen bonds in the sample (Abou Oualid et al., 2017). Broad band ranging from 401 cm^−1^ to 530 cm^−1^, highlighted the red dotted box, can be attributed to the stretching vibration of ZnO (Rohith et al., 2018). Compared to the strong absorption Zn-O peak in the ZnO-NP (Figure 4B), the intensity of Zn-O peak was slightly reduced in the ZnO-Ag-NP, due to the successful binding of Ag on the surface of the nanoparticle (Basavalingiah et al., 2019).

Characteristic peaks in the range of 900 to 1500 cm^−1^ (yellow box) are most probably referring to the presence of oxygen stretching and bending. It might also display the peaks of remaining compounds from the peel extract. All FTIR peaks and the functional group assigned to them are listed in Table 1. Bands found between 1343 and 1392 cm^−1^ corresponds to C=O bending, and peaks at 1529 and 1552 cm^−1^ represent O-H bending vibrations (Satdeve et al., 2019). Sharp peaks between 1529 and 1552 cm^−1^ might also belong to the stretching peak of C=O group present in the polyphenol content of the plant extract, for example gallic acid (Pandiyan et al., 2019). Weak band of C-N group were also detected in the range of 1080 to 1360 cm^−1^ (Das et al., 2016). The Ag^+^ corresponding peak was also detected around 1029 cm^−1^ in ZnO-Ag-NPs (Pandiyan et al., 2019).

**Table 1:** FTIR peaks and corresponding functional groups.

FTIR peak (cm^−1^)	Functional group assigned	References
401–530	Zn-O peak	Rohith et al. (2018)
3332–3385	O-H group	Abou Oualid et al. (2017)
1343–1392	C=O bending	Satdeve et al. (2019)
1529–1552	O-H bending vibrations	Satdeve et al. (2019)
	C=O stretching peak	Pandiyan et al. (2019)
1080–1360	C-N group	Das et al. (2016)
1029	Ag^+^ corresponding peak	Pandiyan et al. (2019)

### 3.4. High resolution transmission electron microscopy analysis

**Figure 5:**
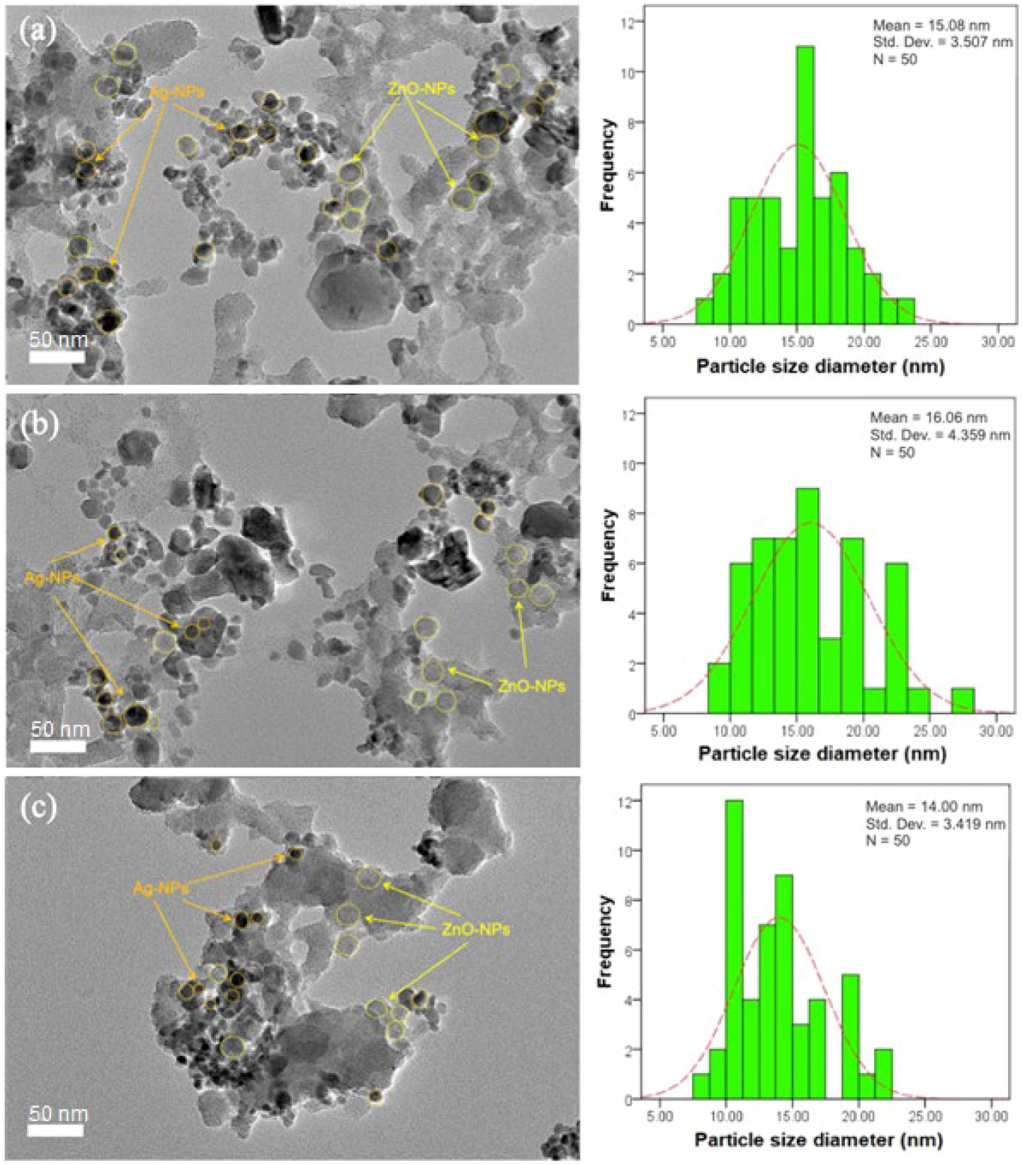
HR-TEM images of ZnO-Ag-NPs biosynthesized in **(A)** pH 4; ZA-4, **(B)** pH 7; ZA-7, and **(C)** pH 9; ZA-9 with their respective particle size distribution histograms.

Morphology and particle distribution in the nanoparticles were assessed by HR-TEM. TEM images (Figures 5A–C) showed the presence of irregular agglomerated particles in the ZnO-Ag-NPs. Ag-NPs were detected as the darker spherical particles with the mean diameter of 15.08, 16.06, and 14.00 nm for ZA-4, ZA-7, and ZA-9, respectively, as shown in the particle distribution histograms. Smaller particle size of the Ag-NPs in the samples prepared at pH 9 (Figure 5C) might be due to the effect of higher concentration of ammonia in the reaction. Several publications have reported the size-controlling role of ammonia in high pH level, as it can help fasten the reaction rate of silver nucleation as well as stabilizing the Ag-NPs (Suwatthanarak et al., 2016; Pimpan and Ritthichai, 2018; Marciniak et al., 2020).

**Figure 6:**
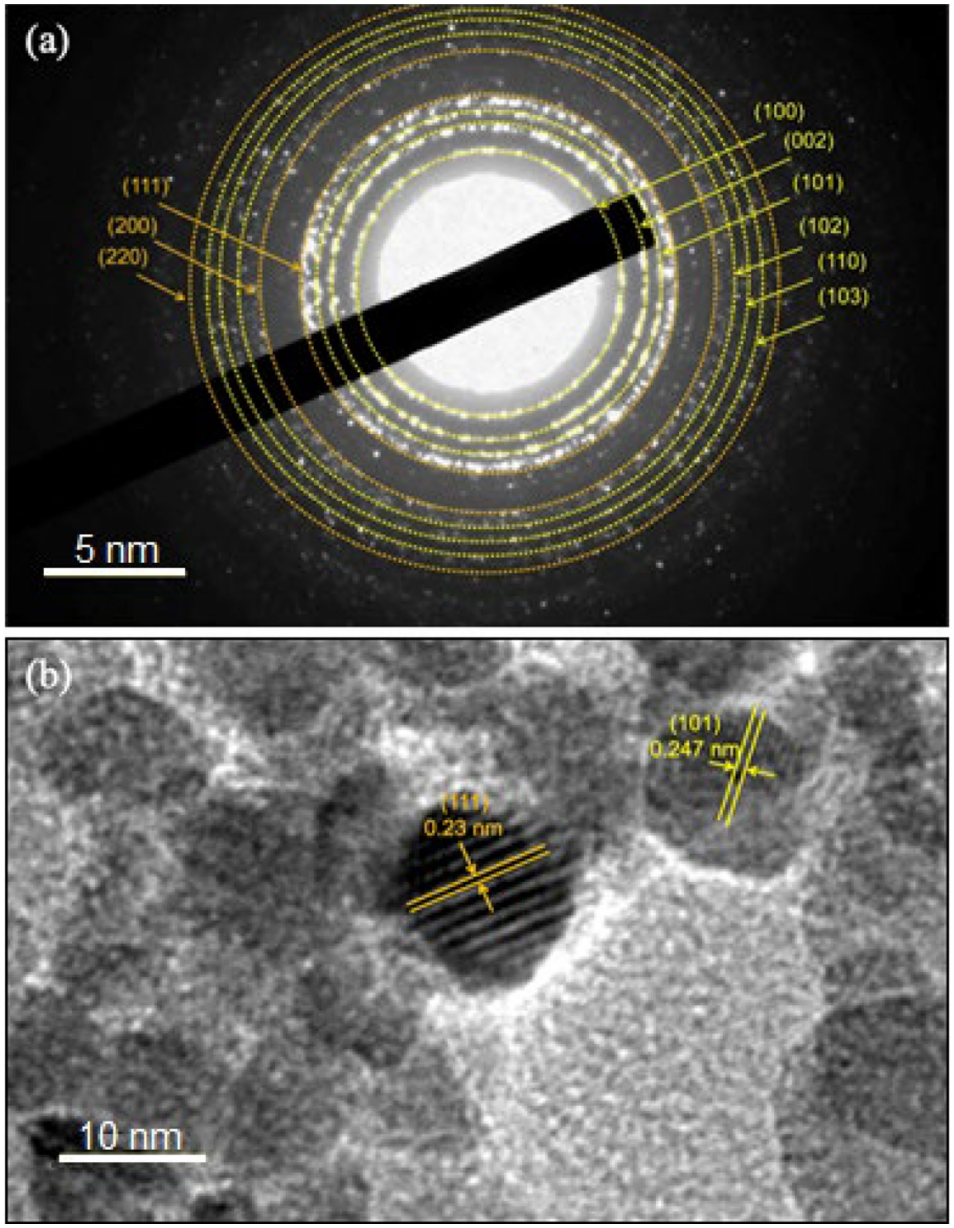
**(A)** Selected area electron diffraction (SAED) pattern and **(B)** d-spacing values in the ZA-9 nanoparticles.

Further characterization of the ZnO-Ag-NPs (ZA-9) using selected area electron diffraction (SAED), as shown in Figure 6A, revealed clear rings indexed to both hexagonal wurtzite phase structure of ZnO (yellow rings) and face centered cubic phase of Ag (orange rings). From the magnified HR-TEM image (Figure 6B), an interplanar lattice spacing value of 0.247 nm, corresponding to the (1 0 1) plane of hexagonal ZnO, was measured. It indicates that one of the growth planes of the ZnO-NPs is along the (1 0 1) plane (Sharma et al., 2018; Marciniak et al., 2020). A d-spacing value of 0.23 nm, corresponding to the *hkl* lattice plane of (1 1 1) of cubic Ag phase (Le et al., 2019), was also determined in the magnified image. These results are consistent and in complete agreement with the XRD results discussed in Figure 2.

**Figure 7:**
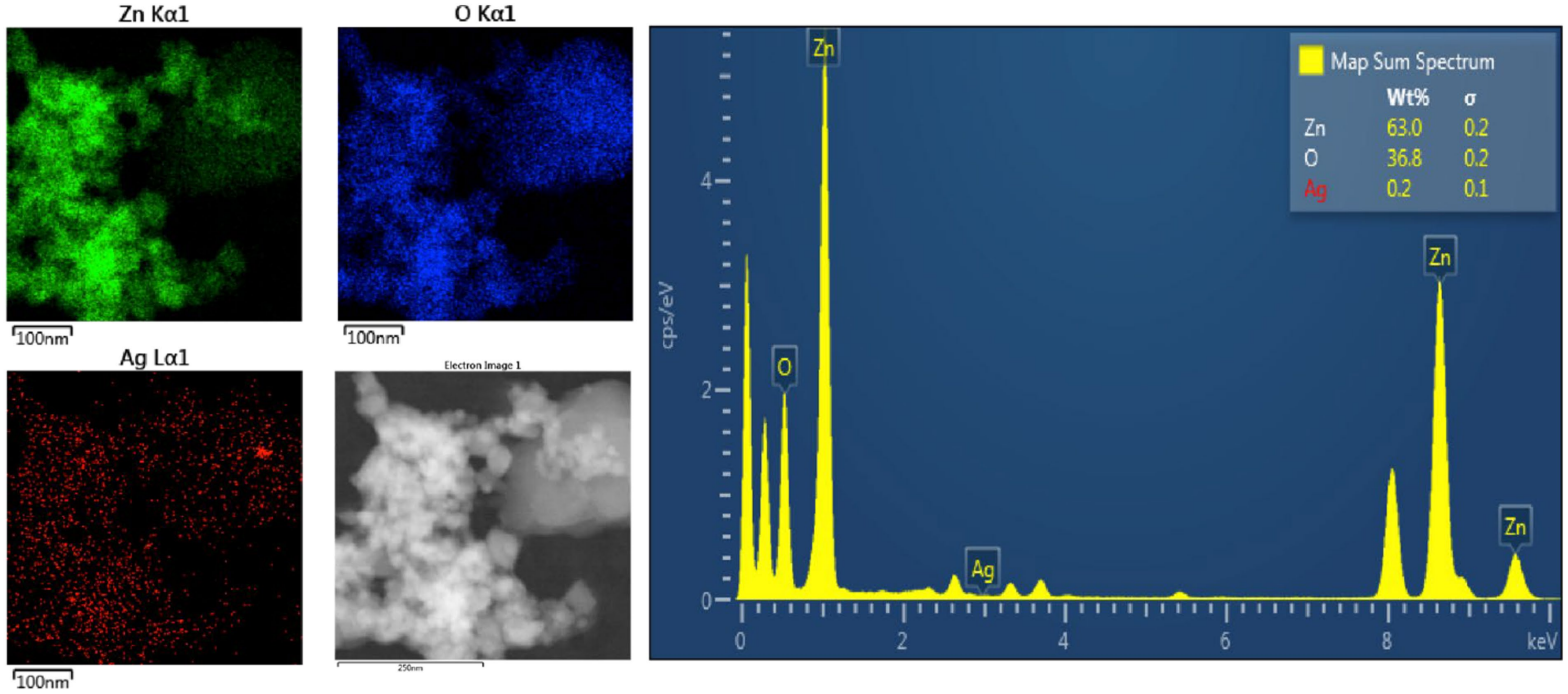
Energy Dispersive X-Ray Spectroscopy spectra showing elemental mapping of ZA-9.

Energy Dispersive X-Ray Spectroscopy (EDS) was also carried out to determine the elemental composition in the ZA-9, where the presence of zinc (Zn), oxygen (O) as well as silver (Ag) elements was detected in the sample (Figure 7). The elemental mapping images revealed uniformly distribution of Ag over the surface of the ZnO-NPs. Quantifiable EDS weight ratios of 63, 36.8, and 0.2% were measured for Zn, O and Ag, respectively, in the sample.

### 3.5. ZnO- and ZnO-Ag-NPs exhibit antibacterial effects

**Figure 8:**
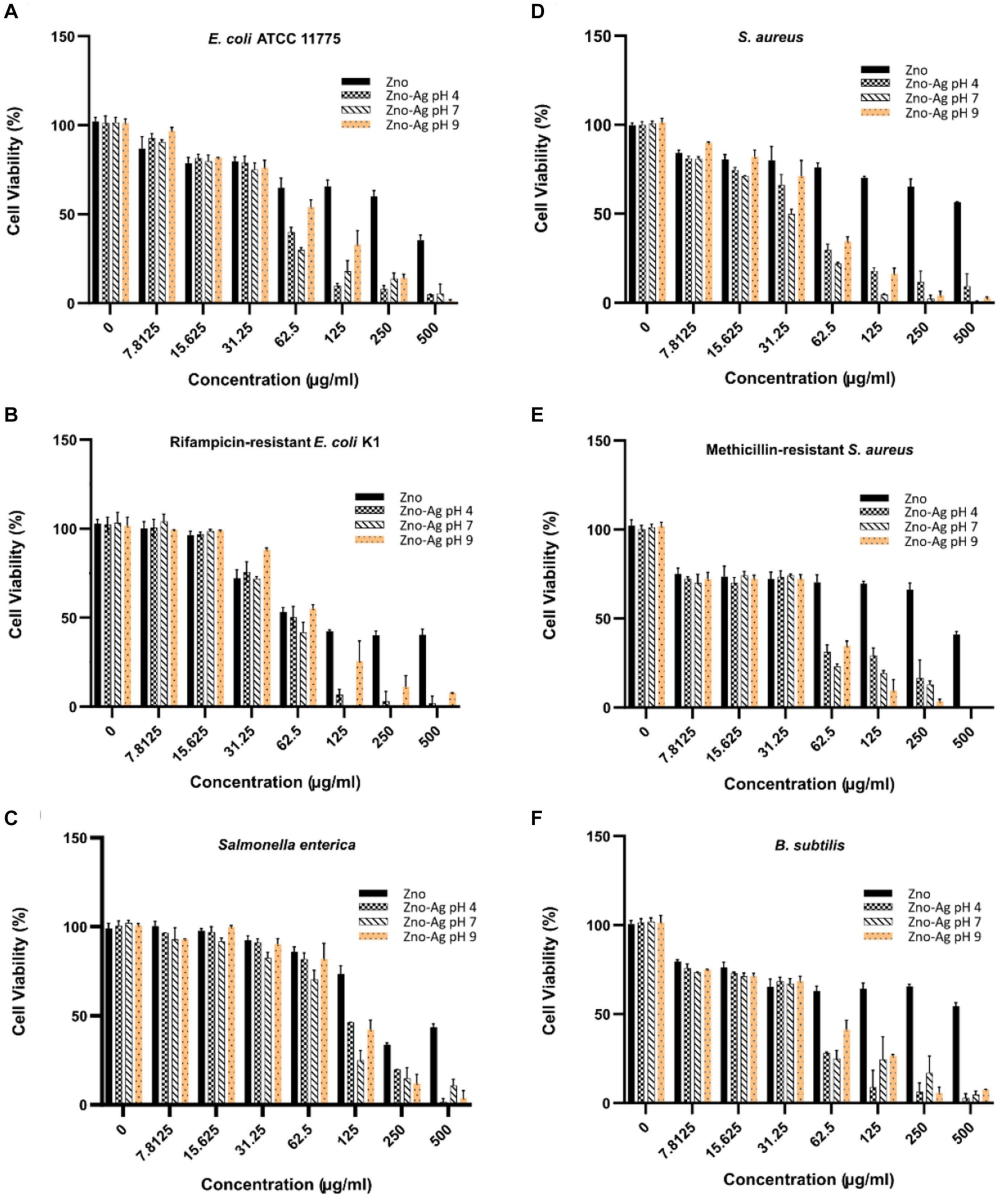
Effects of different concentrations of ZnO- and ZnO-Ag-NPs on the viability of **(A)** *E. coli*, **(B)** *E. coli* K1, **(C)** *S. enterica*, **(D)** *S. aureus*, **(E)** MRSA, and **(F)** *B. subtilis*. Experiments were carried out in triplets; The error bars indicate standard deviations of the means.

ZnO-NPs has been shown to significantly inhibit bacteria growth (Raghupathi et al., 2011). In this study, we investigate if plant-mediated synthesized ZnO and ZnO-Ag-NPs could inhibit bacterial growth. Our data revealed a broad range of antibacterial activities of ZnO-Ag-NPs against both gram-positive and gram-negative bacteria, as shown in Figures 8A–F. The growth of both groups of bacteria was found to be affected by the NPs in a concentration-correlated manner. Significantly less concentration of the ZA-4, ZA-7, and ZA-9 nanoparticles was needed to reach more than 50% inhibition of bacterial growth when compared to ZnO-NP Z-6.

In alignment to the findings presented in Figure 8, calculated MIC_50_ (Table 2) revealed much lower antibacterial efficacy of ZnO-NPs (Z-6) against the bacterial strains when compared to the ZnO-Ag-NPs. The data also showed higher resistance of the tested Gram-positive bacteria toward the pristine ZnO-NPs (Z-6), most probably due to its thicker cell wall, making it harder to permeate (Mthana et al., 2022). Interestingly, the ZnO-Ag-NPs (ZA-4, ZA-7, and ZA-9) showed better antibacterial activities against the Gram-positive strains. This might be due to strong attraction of positively charged Zn^2+^ and Ag^+^ ions dissociated from ZnO-Ag-NPs toward the negatively charged surface of Gram positive bacteria, leading to damage of the cell membrane. ZA-7 displayed the best growth inhibition activity against *S. aureus*, while ZA-4 could remarkably inhibit the growth of *MRSA* and *B. subtilis*. ZA-7 also exhibited the best antibacterial activity against the Gram-negative bacteria, especially *S. enterica*. Higher inhibition activities of ZnO-Ag-NPs could also be contributed by the surface coating by *P. granatum* F. peels extract as they are proven to have antimicrobial properties as well (Singh et al., 2018).

**Table 2:** Minimum concentration of ZnO-NPs and ZnO-Ag NPs inhibiting 50% bacterial growth (MIC_50_).

NPs	MIC_50_ (μg/mL)					
	Gram-negative			Gram-positive		
	*E. coli*	*E. coli K1*	*S. enterica*	*S. aureus*	MRSA	*B. subtilis*
Z-6	387.84 ± 26	30.99 ± 2.51	145.81 ± 7.81	>500	>500	>500
ZA-4	50.00 ± 2.52	60.54 ± 4.84	123.84 ± 5.99	57.88 ± 4.12	32.90 ± 2.11	25.89 ± 1.99
ZA-7	46.37 ± 2.90	50.45 ± 3.21	66.71 ± 3.58	28.29 ± 1.04	44.93 ± 3.42	33.0 ± 2.02
ZA-9	66.65 ± 3.25	67.12 ± 3.68	111.49 ± 4.88	45.01 ± 2.09	49.62 ± 5.22	47.48 ± 3.98

**Figure 9:**
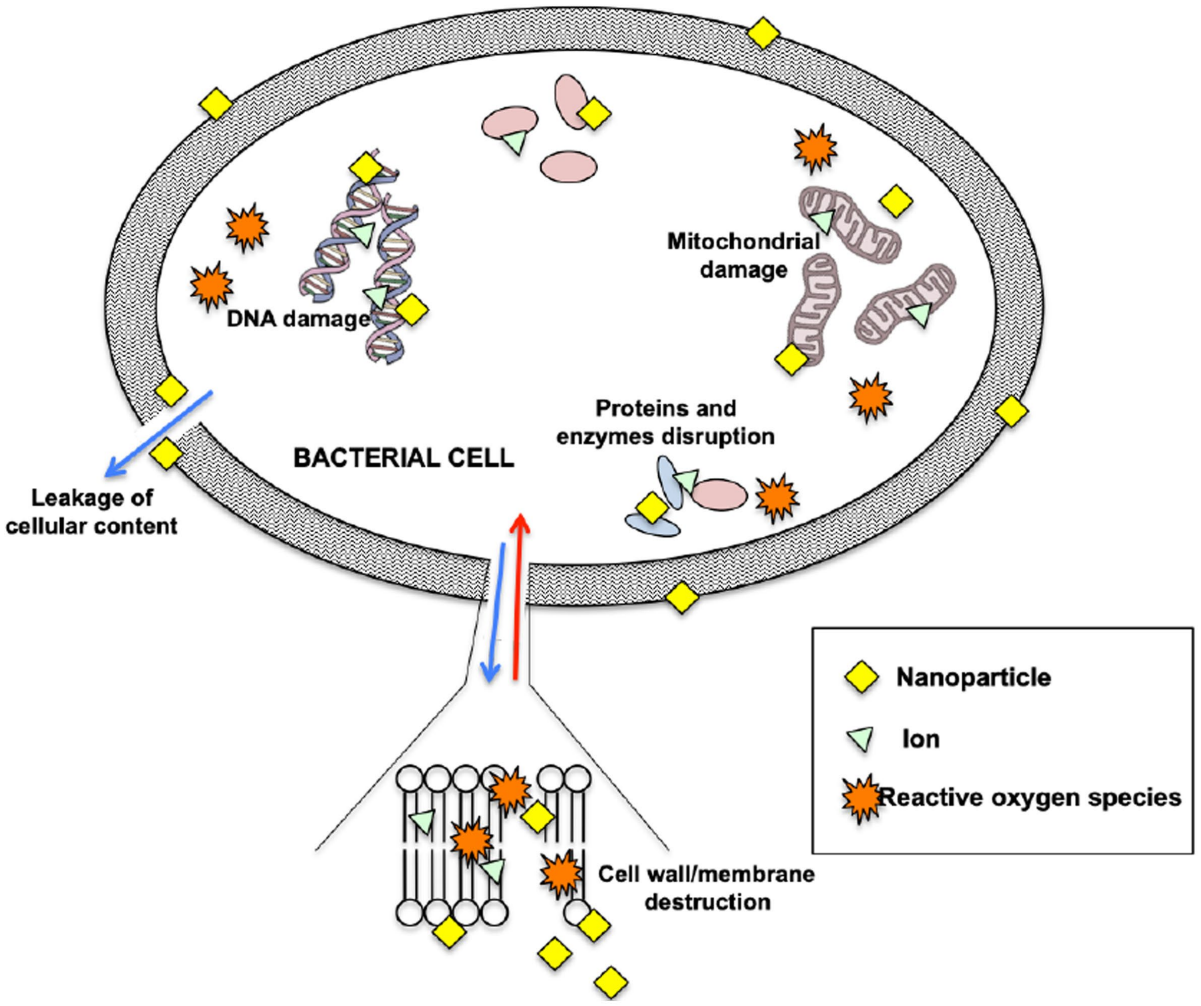
Possible mechanism of antibacterial activity of nanoparticles.

The exact mechanism of killing bacteria by ZnO-NPs and ZnO-Ag-NPs is still not fully understood. Generally, it seems that these NPs directly interact with the bacterial cell wall or membrane by releasing metal ions that disrupt the cell permeability, causing damage to the first line of defense (Gupta et al., 2018). Upon entry into the cells, the NPs and free metal ions will affect the bacteria’s biochemical processes by causing DNA damage and protein denaturation (Khan et al., 2018). This will finally trigger cell death as the bacteria fails to normally replicate. Figure 9 illustrates the possible antibacterial mechanism once in contact with NPs.

The possible explanation for the higher antibacterial activity of ZnO-Ag-NPs as compared to ZnO-NP might be due to structural changes of ZnO-NP after being coupled with Ag; introduction of Ag atoms onto the surface of ZnO-NPs lead to the formation of lower electron state in the band gap which can trap photogenerated charge carriers (Sukri et al., 2021), leading in an increase in electron–hole pairs which could react with oxygen and water to produce reactive oxygen species (ROS), as a critical element in triggering cell death.

### 3.6. Zn-NPs and ZnO-Ag-NPs inhibit proliferation of cancer cells

**Figure 10:**
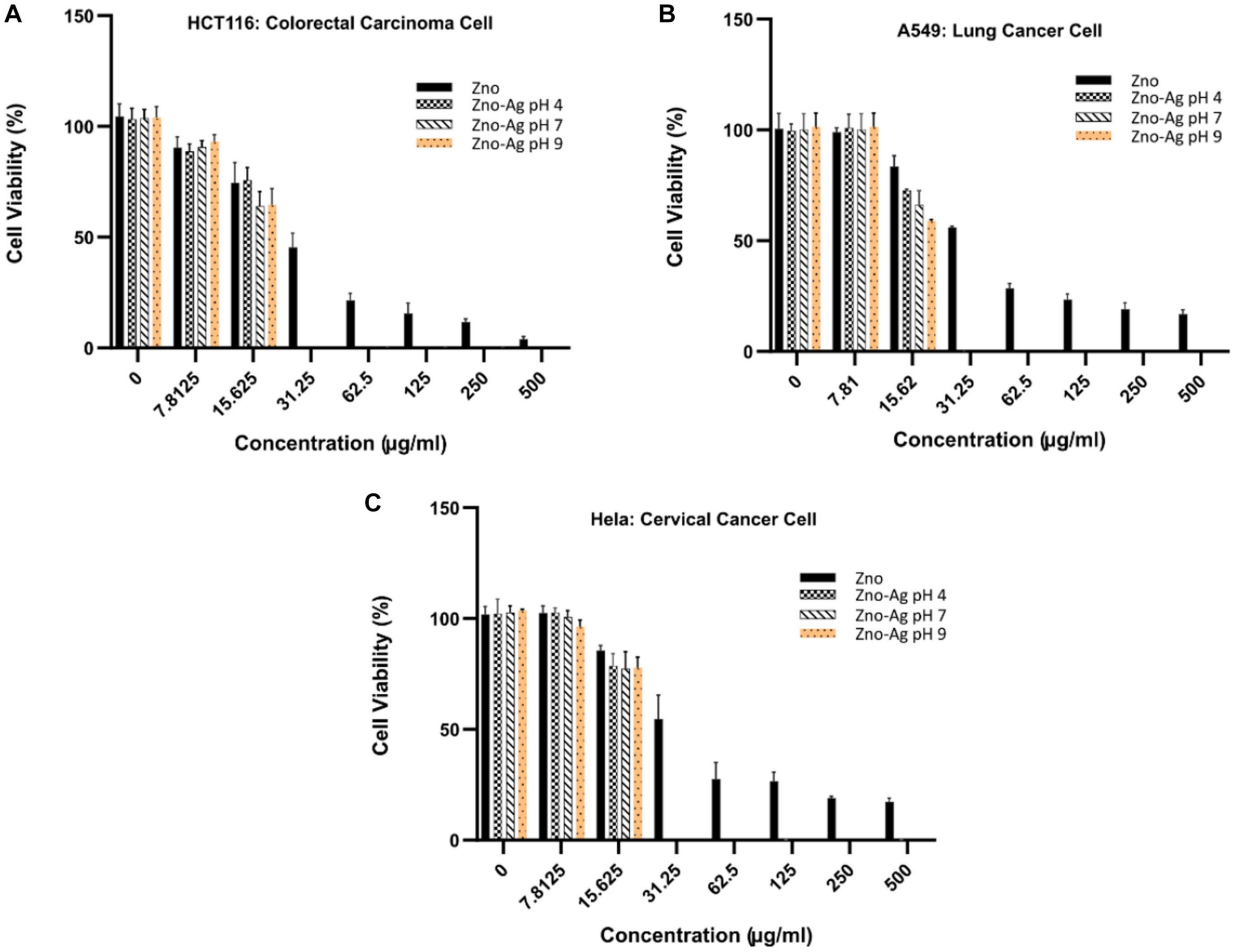
Cytotoxicity effects of ZnO-NPs and ZnO-Ag-NPs on **(A)** colorectal HCT116, **(B)** lung A549, and **(C)** cervical Hela cancer cells. Experiments were carried out in triplets; The error bars indicate standard deviations of the means.

To examine the cytotoxicity effect of the nanoparticles *in vitro*, colorectal (HCT116) lung (A549) and cervical (Hela) cancer cells were treated with different concentrations of the nanoparticles, and anti-proliferative effect was investigated by MTT assay. The results, summarized in Figure 10, showed that the nanoparticles could dose-dependently inhibit the proliferation of the cells. We found that the ZnO-Ag-NPs (ZA-4, ZA-7, and ZA-9) had significantly better anti-proliferative effect on cancer cells when compared to the ZnO-NP (Z-6), indicating the enhancement of cytotoxic activities of ZnO-NPs after conjugation with Ag. In contrast to the pristine ZnO-NPs, the ZnO-Ag-NPs could completely inhibit cell growth at the low concentration of 31.25 μg/mL. Based on the calculated IC_50_ values, presented in Table 3, ZnO-Ag-NPs exhibited notably higher cytotoxic activities against the cancer cells 72 h after the treatment when compared to ZnO-NP. The best anti-proliferative effect was observed in the ZA-9-treated cells with the lowest IC_50_ values of 15.12–16.11 μg/mL toward the cancer cells. This may be related to the smaller size of ZA-9 as compared to ZA-4 and ZA-7, leading to increased surface chemistry of the NPs with cancer cells (Mthana et al., 2022).

**Table 3:** Inhibitory concentration killing 50% cells (IC_50_) of ZnO-NPs and ZnO-Ag NPs against cancer cells.

Samples	IC_50_ (μg/mL)		
	HCT116 cells	A549 cells	Hela cells
Z-6	28.89 ± 1.63	34.39 ± 2.19	33.77 ± 2.23
ZA-4	17.45 ± 1.01	17.06 ± 1.21	17.1 ± 0.98
ZA-7	15.46 ± 1.14	16.5 ± 0.89	17.18 ± 1.68
ZA-9	15.12 ± 1.42	15.89 ± 1.11	16.11 ± 1.75

Similar to antibacterial mechanism, Zn^2+^ ions and free radicals released from Ag have been reported to induce ROS production, triggering cells death via ROS-mediated apoptotic process. An interesting study from Sachdev et al. (2015) investigated the cellular uptake of carbon dots-silver@zinc oxide nanocomposite (CD Ag@ZnO NC) inside cancer cells, revealing cytoplasm and nuclear localization of the NCs in a dose-dependent manner. In lower concentration of 20 μg/mL, the NC was found mostly in the cytoplasm whereas nuclear localization was also observed in higher concentrations of 50 and 70 μg/mL.

## 4. Conclusion

In conclusion, our study successfully synthesized ZnO-Ag-NPs using a green synthesis approach with *P. granatum* (pomegranate) fruit peels extract under varying pH conditions. XRD analysis confirmed the crystalline structure of the NPs and showed increased crystallinity of ZnO-Ag-NPs in higher pH levels. UV-vis spectroscopy and FTIR analysis confirmed the incorporation of Ag onto the ZnO-NPs as they showed shifting of absorption peaks toward 420–430 nm as well as characteristic Zn-O bond with reduced intensity between 401 and 530 cm^−1^, respectively. From HR-TEM, the synthesized Ag-NPs were shown to be spherical in shape with a mean size of around 14–16 nm and were uniformly distributed on the ZnO matrix. The ZnO-Ag-NPs exhibited significantly higher antibacterial activity against both Gram-positive and Gram-negative bacteria than pristine ZnO-NPs. Furthermore, the ZnO-Ag-NPs showed enhanced cytotoxic effects against colorectal-, lung-, and cervical cancer cells compared to ZnO-NPs, indicating their potential as therapeutic nanomaterials for cancer treatment. Our study highlights the potential of green-synthesized ZnO-Ag-NPs for various biomedical applications, and further research can be conducted to explore the surface modification of NPs for more efficient targeting of bacteria and cancer cells.

## Data availability statement

The original contributions presented in the study are included in the article/supplementary material, further inquiries can be directed to the corresponding author.

## Author contributions

SM, KS, S-YT, JC, L-TO, ML-KS, NI, and HM: conceptualization and writing—review and editing. SM, KS, S-YT, JC, L-TO, ML-KS, and NI: methodology. SM, KS, and S-YT: project implementation and formal analysis. KS, S-YT, and HM: validation and visualization. KS, S-YT, JC, and L-TO: investigation. KS and S-YT: resources, supervision, and funding acquisition. SM and KS: writing—original draft preparation. KS: project administration. All authors have read and agreed to the published version of the manuscript.

## Funding

KS and SM gratefully acknowledge support from the Takasago Thermal Engineering Co., Ltd. (grant number: R.K.130000.7343.4B422) and Malaysia–Japan International Institute of Technology (MJIIT).

## Conflict of interest

The authors declare that the research was conducted in the absence of any commercial or financial relationships that could be construed as a potential conflict of interest.

## Publisher’s note

All claims expressed in this article are solely those of the authors and do not necessarily represent those of their affiliated organizations, or those of the publisher, the editors and the reviewers. Any product that may be evaluated in this article, or claim that may be made by its manufacturer, is not guaranteed or endorsed by the publisher.

## Footnotes

^1^https://www.aatbio.com/tools/ic50-calculator
